# Neutral sphingomyelinases restrict natural killer cells activity against lung cancer

**DOI:** 10.1007/s00262-026-04393-0

**Published:** 2026-04-22

**Authors:** Riccardo Cinotti, Mannon Geindreau, Anna Bergqvist, Ying Yang, Andreas Lundqvist

**Affiliations:** Department of Oncology-Pathology, Karolinska Institutet, Stockholm, Sweden

**Keywords:** Natural killer cells, Lung adenocarcinoma, Sphingomyelin, Immunotherapy

## Abstract

High frequency and activity of tumor-infiltrating natural killer (NK) cell is associated with improved prognosis in several solid cancers including non-small cell lung cancer. However, multiple factors can suppress NK cell activity within the tumor microenvironment, resulting in impaired killing of tumor cells. For NK cells to engage and kill target cells, stabilization of lipid rafts is essential. Sphingomyelin is involved in lipid raft formation and is catabolized by sphingomyelinases. While inhibition of sphingomyelinases enhances NK cell-mediated killing of tumor cells, it is unknown how sphingomyelinases impact on the ability of NK cells to infiltrate solid tumors. Here we demonstrate that inhibition of sphingomyelinases results in increased activation and ability of NK cells to infiltrate lung adenocarcinoma. Overall, our findings support the use of sphingomyelinase inhibitors as enhancers of NK cell activation.

**Supplementary Information:**

The online version contains supplementary material available at 10.1007/s00262-026-04393-0.

## Introduction

Lung cancer is the primary cause of cancer-related mortality. In non-small cell lung cancer (NSCLC), which consists of lung adenocarcinoma (LUAD) and squamous cell carcinoma (LUSC), the frequency of tumor-infiltrating natural killer (NK) cell is associated with improved prognosis [1, 2]. NK cells are innate lymphocytes that display numerous membrane surface protrusions allowing a direct contact with tumor cells. Membrane fluidity and cytoskeleton remodeling impact on the movement and stabilization of lipid rafts at the immunological synapse which is essential for NK cell-mediated killing of target cells [3]. Sphingomyelin (SM) is a sphingolipid present in all cellular membranes and plasma lipoproteins. Its synthesis from ceramide (CER) and phosphatidylcholine is catalyzed by sphingomyelin synthases (SMSs). In contrast, sphingomyelinases catabolize SM and are divided into neutral (NSMs) and acid (ASMs) [4]. ASMs are encoded by the same gene (*SMPD1*), whereas NSMs (NSM1-3) are, respectively, encoded by three distinct genes (*SMPD2*, *SMPD3*, and *SMPD4*).

NSM3 (*SMPD4*) is part of the TNFα receptor type 1 (TNFR1) and the factor associated with NSM activation (FAN) signaling pathway [5]. In the TNFR1-FAN-NSM3 pathway, following activation of TNFR1 by TNFα, receptor-associated FAN activates the caspase cascade and NSM3-mediated conversion of SM to CER leading to apoptosis and cytoskeleton remodeling via the cell division control protein 42 (cdc42) [6]. Additionally, CER itself is a known pro-apoptotic molecule and precursor of sphingosine-1-phosphate (S1P), a key mediator within the TME that affects mitochondrial respiration and cytochrome c oxidase assembly [7].

Although NSMs can mediate TNFα-induced apoptosis in cardiac tissue [8], their role in immune cell activity is largely unknown. A recent study demonstrated that intratumoral NK cells have an altered surface morphology compared with peripheral blood NK cells in which protrusions and lytic immune synapses are significantly reduced or absent [9]. Notably, several SM isoforms were decreased in intratumoral NK cells compared with peripheral blood NK cells resulting in an altered SM metabolism [9]. However, whether SM impacts on the ability of NK cells to infiltrate tumors is currently unknown.

Here we investigated whether targeting NSMs will modulate NK cell-mediated tumor killing and phenotype as well as tumor infiltration in lung adenocarcinoma. Overall, inhibition of NSMs resulted in increased activity by NK cells. Furthermore, NK cells displayed a higher capacity to infiltrate lung adenocarcinoma tumors upon inhibition of NSMs. These results extend our current knowledge on the role of NSMs in anti-tumor immune responses and warrants for modulating the SM metabolism to develop NK cell-based therapies against solid cancers.

## Materials & methods

### Cell culture

Lung adenocarcinoma A549, H23, and H1975 cells were maintained in RPMI 1640 + Glutamax (Gibco) supplemented with 10% FBS (Gibco), and 1% penicillin–streptomycin (Gibco). For spheroid cultures, 2 × 10^4^ A549 cells/well were seeded in a 96-well ULA Nunclon® plate (Thermo Fisher) in DMEM/F12 (Gibco, 10% FBS, and 1% PS) and incubated for 2–5 days. Peripheral blood mononuclear cells (PBMCs) were isolated by Ficoll-Paque (Cytiva AB) centrifugation from healthy donors. NK cells were isolated from PBMCs using an NK cell isolation kit (Miltenyi Biotec) and cultured in X-VIVO 15 (Lonza) supplemented with 10% hAb (Karolinska Blood Bank), 1% PS, and 0.3 μg/mL IL-2 (Novartis). The NSM inhibitor GW4869 (MedChem Express) was used at 1 µM for 24–72 h of treatment.

### Flow cytometry

Following treatment, NK cells were washed twice in PBS and thereafter stained for viability (Live/Dead Aqua dye at 1:1000 or NIR 1:1000) in PBS for 20 min at 4 °C. After an additional two washes with FACS buffer (5% FBS in PBS), cells were stained for surface markers for 20 min at 4 °C (Supplementary Table 1). Cells were then washed once with permeabilization buffer, fixed, and permeabilized (FoxP3 Perm/Fix kit, Invitrogen) for 20 min at room temperature and stained for intracellular markers for 30 min at 4 °C. Cells were analyzed by flow cytometry (NovoCyte Quanteon). Results were analyzed in FlowJo (v10.10.0 for Windows).

### NK cell cytotoxicity and proliferation

NK cell cytotoxicity was evaluated in monolayer by chromium release assay [10] and flow cytometry and in tumor spheroids by real-time live-cell imaging. For spheroid killing in Incucyte SX5, NIR Cytotox dye (Sartorius, 1:4000) was added at the beginning of the 72 h co-culture with NK cells. To evaluate NK cell proliferation, 1 × 10^5^ freshly isolated CFSE-labeled cells were resuspended in X-VIVO 15 supplemented with 10% hAb, 1% PS, and 1 μg/mL IL-2 with or without GW4869. Cells were incubated at 37 °C in a 96-well flat-bottom (Thermo Fisher) plate for 7 days and analyzed by flow cytometry.

### Tumor infiltration by NK cells

On day 5 of tumor spheroid culture, CFSE-labeled NK cells were added at 3:1 ratio in DMEM/F12. GW4869 was either added during the co-culture or as a pre-treatment of NK cells for 24 h. For pre-treatment, NK cells were washed with PBS prior to co-culture. On day 8, spheroids were washed twice with PBS before spheroid dissociation using TrypLE Express (Gibco). Finally, the single-cell suspension was analyzed by flow cytometry as described above.

### TCGA and database analysis

TCGA data were retrieved from the Human Protein Atlas (THPA, https://www.proteinatlas.org/) [11], GEPIA and TIMER2 querying for *SMPD1*, *SMPD2*, *SMPD3*, and *SMPD4*. In TIMER2 infiltration, results were corrected for purity. Results were analyzed in Prism (v10 for Windows). THPA was used to retrieve data for the expression of SMPDs in PBMCs from Monaco et al. [12].

### Statistical analysis

Statistical analysis was performed on Prism (v10 for Windows), and applied statistical tests are indicated in each figure legend. Normality was assessed for each dataset. In each figure, *p*-values are indicated as significance levels (* *p* < 0.05, ** *p* < 0.01, *** *p* < 0.005, and **** *p* < 0.0001) or numeric values.

## Results

### Low SMPD4 and high NK cell infiltration is prognostic in patients with lung adenocarcinoma

**Fig. 1:**
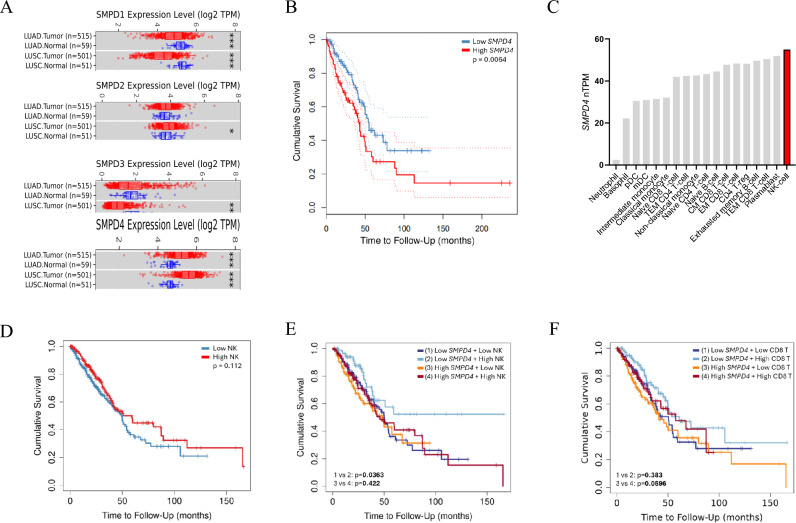
Low SMPD4 and high NK cell infiltration are prognostic in patients with lung adenocarcinoma. **A** Expression of SMPDs in lung adenocarcinoma (LUAD) and squamous cell lung carcinoma (LUSC) compared to normal tissue. **B** Survival analysis of LUAD patients stratified in high (*n* = 120) and low (*n* = 120) SMPD4 expression quartiles extracted from GEPIA. **C** Expression in normalized transcripts per million (nTPM) of SMPD4 in peripheral blood mononuclear cells (PBMC) extracted from The Human Protein Atlas (Monaco dataset). **D** Survival analysis of LUAD patients (1–200 months) stratified based on average NK cell gene expression (QUANTISEQ), corrected for sample purity (*n* = 482). **E** Survival analysis of LUAD patients (1–200 months) stratified based on average expression of SMPD4 and NK cell infiltration (QUANTISEQ), corrected for sample purity (*n* = 482). **F** Survival analysis of LUAD patients (1–200 months) stratified based on average expression of SMPD4 and CD8 T cell infiltration (QUANTISEQ), corrected for sample purity (*n* = 482). Data from **A**, **D**, **E**, and **F** were extracted from TIMER2

Since sphingolipid metabolism is increasingly recognized as a key pathway in tumor progression, immune evasion, and response to cancer immunotherapy, the expression of sphingomyelinases was investigated across normal tissues and cancers. In general, *SMPD1* (ASM) and *SMPD3* (NSM2) were lower, whereas the expression of *SMPD2* (NSM1) and *SMPD4* (NSM3) was higher in tumor compared with normal tissues (Supplementary Fig. 1A). Similarly, the expression of *SMPD1* and *SMPD4* was lower and higher in LUAD compared with normal tissue, respectively (Fig. 1A). Furthermore, *SMPDs* were associated with poor prognosis across several cancers including LUAD, adrenocortical carcinoma (ACC), kidney renal papillary cell carcinoma (KIRP), low-grade glioma (LGG), liver hepatocellular carcinoma (LIHC), mesothelioma (MESO), and prostate adenocarcinoma (PRAD) (Fig. 1B and Supplementary Fig. 1B). Among leukocytes, NK cells showed the highest expression of *SMPD4* (Fig. 1C). Also, *SMPD4* expression was higher compared with *SMPD1-3* in NK cells while other PBMC populations expressed *SMPD1-3* at higher levels than NK cells (Fig. 1C and Supplementary Fig. 1C, D). In LUAD, NK cell gene expression alone did not influence survival (Fig. 1D). Likewise, NK cell gene expression did not influence survival in patients with *SMPD4* high LUAD tumors. In contrast, overall survival was longer in patients with *SMPD4* low LUAD tumors and high NK cell gene expression (Fig. 1E). No difference in patient survival was observed between CD8 T cell high and low gene expression regardless of *SMPD4* expression (Fig. 1F). These results show that tumors express varying levels of sphingomyelinases and that low *SMPD4* expression and high NK cell infiltration is associated with longer overall survival in patients with LUAD.

### Inhibition of NSMs results in activation of NK cells

**Fig. 2:**
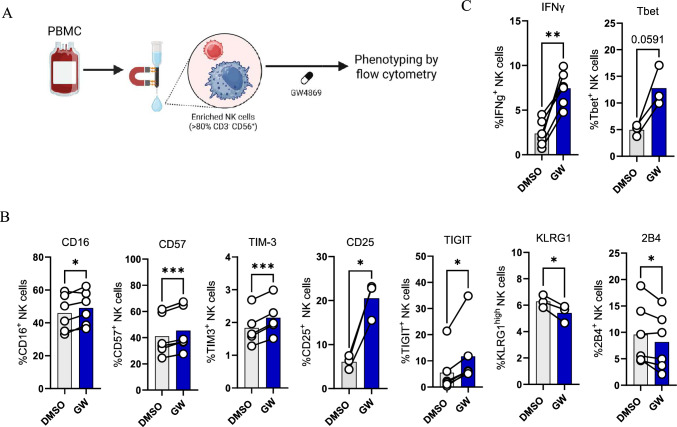
Inhibition of NSMs results in activation of NK cells. **A** NK cells were enriched by magnetic column separation from PBMC and treated for 24 h with 1 μM GW4869 (GW) and thereafter phenotypically assessed by flow cytometry. Expression of CD16 (*n* = 6), CD57 (*n* = 6), CD25 (*n* = 3), TIGIT (*n* = 6), TIM-3 (*n* = 5), KLRG1 (*n* = 3), and 2B4 (*n* = 6) in NK cells. **C** Intracellular expression of IFNγ (*n* = 5) and Tbet (*n* = 3) in NK cells. Normality was assessed for **B** and **C** by Kolmogorov–Smirnov and Shapiro–Wilk test. Statistical analysis was performed as paired t-tests or Wilcoxon tests for normally and non-normally distributed data, respectively. Biological replicates were collected from at least two independent experiments and values within brackets report the number of total individual NK cell donors

Since *SMPD4* expression and NK cell infiltration influence prognosis in LUAD, NK cells were investigated for their phenotype and activity upon inhibition of NSMs using GW4869 (Fig. 2A). While no difference in proliferation or viability was observed (Supplementary Fig. 3A), the expression of membrane CD16, CD57, CD25, TIGIT, and TIM-3 was significantly increased, whereas the expression of KLRG1 and 2B4 was significantly reduced upon treatment with the NSM inhibitor GW4869 (Fig. 2B). Several other NK cell markers did not change upon treatment with GW4869 (Supplementary Fig. 3B). A modest but significant increase in CD57 expression was observed upon prolonged (48–72 h) treatment with GW4869. However, the viability was significantly reduced upon 48–72-h treatment with GW4869 (Supplementary Fig. 3C). With regard to the production of cytokines, a higher frequency of IFNγ-positive NK cells was observed upon treatment with GW4869. Along these lines, GW4869 treatment resulted in a higher frequency of T-bet-positive NK cells (Fig. 2C). In contrast, the frequency of GM-CSF-positive NK cells reduced after treatment with GW4869, whereas the frequency of IL-10- and IL-6-positive NK cells remained unchanged (Supplementary Fig. 3D). These results show that inhibition of NSMs using GW4869 modulates the expression of cell surface markers and increases IFNγ production by NK cells.

### Inhibition of NSMs increases NK cell cytotoxicity and tumor infiltration

**Fig. 3:**
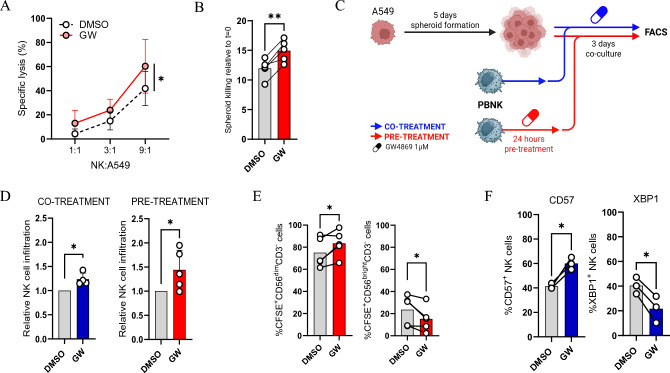
Inhibition of NSMs increases NK cell cytotoxicity and tumor infiltration. **A** Killing of A549 cells co-cultured at effector/target ratio of 9:1 to 1:1 with peripheral NK cells pre-treated for 24 h with GW4869 1 μM, measured by chromium release assay (*n* = 5). **B** Spheroid killing at 72 h relative to *t* = 0 using pre-treated NK cells (*n* = 5). **C** Experimental setup for the evaluation of peripheral NK cells infiltration, either pre-treated or treated during the co-culture, into A549 tumor spheroids. **D** Infiltration relative to DMSO of NK cells pre-treated (*n* = 5) or co-treated with A549 cells (*n* = 5). Mean NK cell infiltration of co-treated cells was 25.7 and 32.2% in the DMSO- and GW-treated groups, respectively. Mean NK cell infiltration of pre-treated NK cells was 36.0 and 49.6% in the DMSO- and GW-treated groups, respectively. **E** Percentage of CD56^bright^ and CD56^dim^ among infiltrated, pre-treated, NK cells (*n* = 5). **F** Expression of surface CD57 and intracellular XBP1 in infiltrated NK cells one day post co-culture (*n* = 3). Normality was assessed by Kolmogorov–Smirnov and Shapiro–Wilk test. Statistical analysis was performed as unpaired or paired t-test, or two-way ANOVA. Biological replicates were collected from at least two independent experiments and values within brackets report the number of total individual NK cell donors

Given the altered phenotype of NK cell upon inhibition of NSMs, the tumor-killing potential of NK cells was evaluated upon treatment with GW4869. GW4869-treated NK cells showed increased killing of A549 cells compared with untreated NK cells in monolayer and spheroids (Fig. 3A, B). When added to A549 tumor spheroids together with NK cells or upon pre-treatment of NK cells, GW4869 induced an increased NK cell infiltration (Fig. 3C, D). In addition, GW4869 treatment resulted in enriched infiltration of CD56^dim^ NK cells, as well as a higher frequency of CD57-positive and reduced frequency of XBP1-positive NK cells (Fig. 3E, F). On the other hand, treatment of NK cells with GW4869 did not affect the proportion of CD56^dim^ and CD56^bright^ in monoculture (Supplementary Fig. 3E). Likewise, GW4869 pre-treatment increased killing and infiltration in H23 spheroids but not H1975 spheroids (Supplementary Fig. 3F, G). These results show that inhibition of NSMs using GW4869 increases NK cell cytotoxicity and infiltration into lung cancer spheroids.

## Discussion

The frequency of tumor-infiltrating NK cell has been associated with improved prognosis in NSCLC [1, 2]. However, NSCLC-infiltrating NK cells display a dysfunctional phenotype [13, 14]. Besides the altered expression of activating receptors compared with peripheral blood NK cells, tumor-infiltrating NK cells have fewer membrane protrusions resulting in reduced formation of lytic immunological synapses and capacity to engage and kill target cells. In addition, tumor-infiltrating NK cells have altered SM content and targeting sphingomyelinase increases cytolytic activity by tumor-infiltrating NK cells [9]. Here we extend these observations showing that NSM inhibition promotes NK cell infiltration in NSCLC.

Few studies have investigated the activity of sphingomyelinases in immune cells. ASMs play a major role in innate immunity where their activity is linked to macrophage-mediated clearance of pathogens, and apoptosis of dendritic cells and recruitment of neutrophils [15–17]. Inflammatory responses induced by TNFα have also been reported to be regulated by NSMs in myeloid cells [18]. In T cells, the activity of NSMs is important in recruitment and migration [19]. However, the role of NSMs in NK cells is largely unknown. A recent study by Zheng et al. reported that restoration of SM levels via inhibition of NSMs enhances cytotoxic activity in tumor-infiltrating NK cells [9]. Here we extend these findings showing that NK cells express higher levels of *SMPD4* compared with other sphingomyelinases and that low *SMPD4* expression and high NK cell infiltration is associated with longer overall survival in patients with LUAD. Inhibition of NSMs resulted in increased expression of TIGIT, TIM-3, KLRG1, and CD57 indicating a mature and exhausted phenotype by NK cells [20, 21]. Yet, NK cells still retained their proliferative capacity and produced even higher levels of IFNγ upon NSM inhibition. Furthermore, NK cells expressed higher levels of CD25 upon NSM inhibition, suggesting maintained activity in the context of regulatory T cell-mediated suppression [22]. Prolonged inhibition of NSMs resulted in reduced NK cell viability supporting a shorter exposure to NSM inhibiting agents.

While adoptive infusion of NK cells shows promise in patients with acute myeloid leukemia, NK cell-based therapies are so far less efficacious in patients with solid cancers, due to inadequate tumor infiltration and persistence in the tumor microenvironment [23, 24]. A recent meta-analysis and systematic review in patients with NSCLC revealed comparable disease control and 1-year overall survival between controls and patients receiving NK cell therapy [25]. Here we show that inhibition of NSM increases NK cell infiltration into NSCLC tumors. Specifically, CD56^dim^ NK cells showed preferentially infiltration. Notably, NSCLC patients with high levels of CD56^dim^ NK cells show prolonged overall survival [26]. Furthermore, a reduced frequency of XBP1-positive NK cells was observed upon inhibition of NSMs. Since XBP1 has been shown to enhance effector functions and survival in NK cells, it would potentially be disadvantageous to inhibit NSMs in NK cells [27]. Nevertheless, the role of XBP1 in NK cells is somewhat unclear since chronic endoplasmic reticulum stress in can hyperactivate XBP1, potentially leading to NK cell dysfunction or exhaustion. In conclusion, our results support that targeting the sphingomyelin pathway represents a valuable approach to increase NK cell activation and tumor infiltration. Studies using patient-derived model systems are warranted to support our findings and to ultimately improve adoptive NK cell therapy.

## Supplementary Information

Below is the link to the electronic supplementary material.

Supplementary file1 (PDF 911 KB)

## Data Availability

All data supporting the findings of this study are available within the paper and its Supplementary Information.
