# Supplementary material for: Neutral sphingomyelinases restrict natural killer cells activity against lung cancer
Source: Cancer Immunol Immunother. 2026 Apr 22;75(5):155. doi: 10.1007/s00262-026-04393-0 (PMC13102996; doi:10.1007/s00262-026-04393-0)
Supplement: Supplementary file 1 — Supplementary file1 (PDF 911 KB) [file 262_2026_4393_MOESM1_ESM.pdf]

SUPPLEMENTARY MATERIAL FOR:

Neutral sphingomyelinases restrict natural killer cells activity against lung cancer

Riccardo Cinotti[1], Mannon Geindreau[1], Anna Bergqvist[1], Ying Yang[1], and Andreas Lundqvist[1]

[1]Department of Oncology-Pathology, Karolinska Institutet, Stockholm, Sweden.

Correspondence: andreas.lundqvist@ki.se

Supplementary Figure 1

Supplementary Figure 2

Supplementary Figure 3

Supplementary Table 1

Supplementary figure 1.

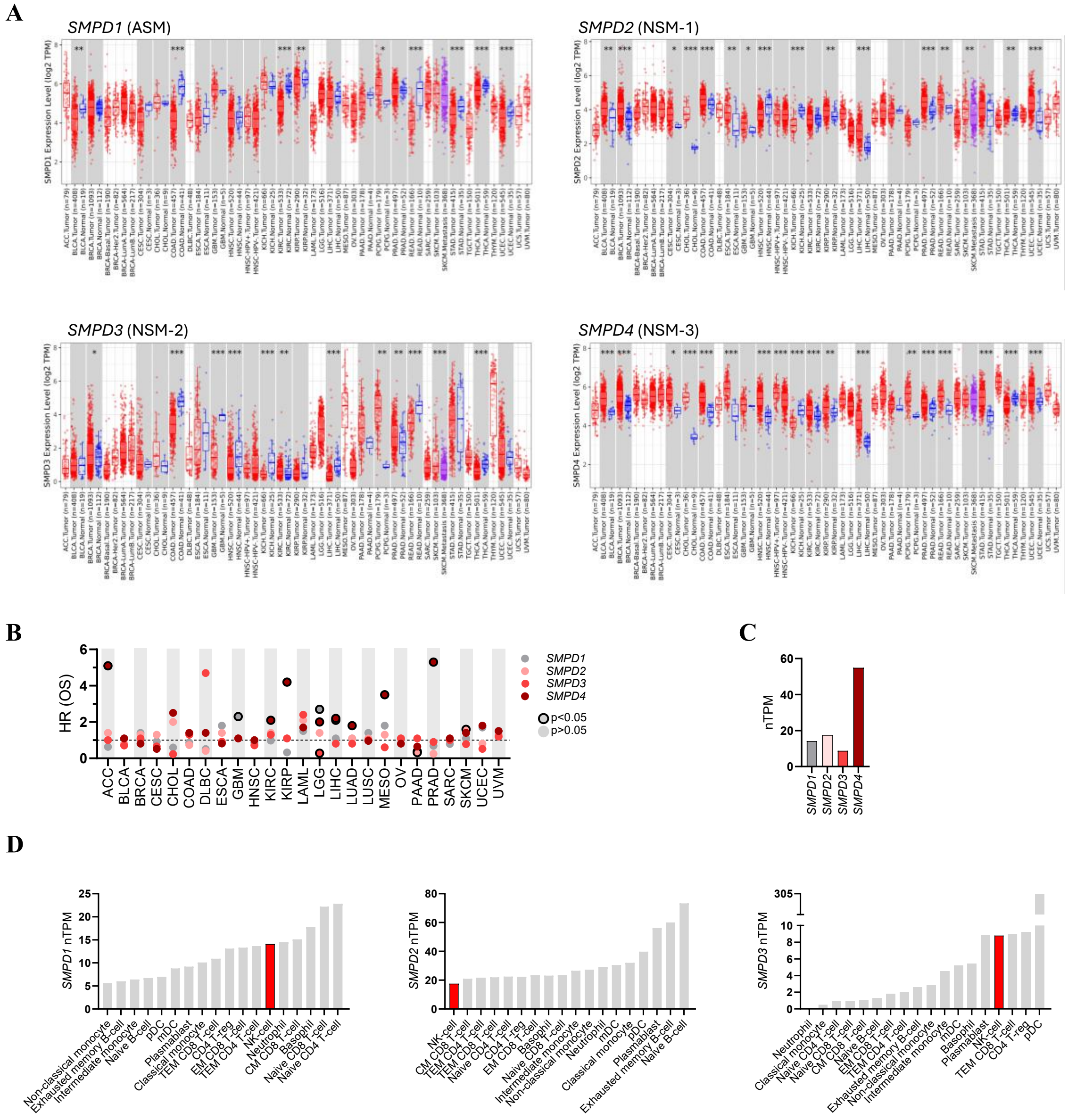

Supplementary figure 1. *(A) Expression of SMPDs in cancers compared to normal tissues, extracted from TIMER2. (B) Hazard ratio (HR) associated to high SMPD expression in selected cancers, where a higher HR(OS) indicates an increased risk of death and a black border indicates a significant result (p<0.05), extracted from TIMER2. (C) SMPD expression in peripheral blood NK cells extracted from The Human Protein Atlas (Monaco dataset). (D) Expression of SMPD1-3 across healthy donor PBMC extracted from The Human Protein Atlas (Monaco dataset).*

Supplementary figure 2.

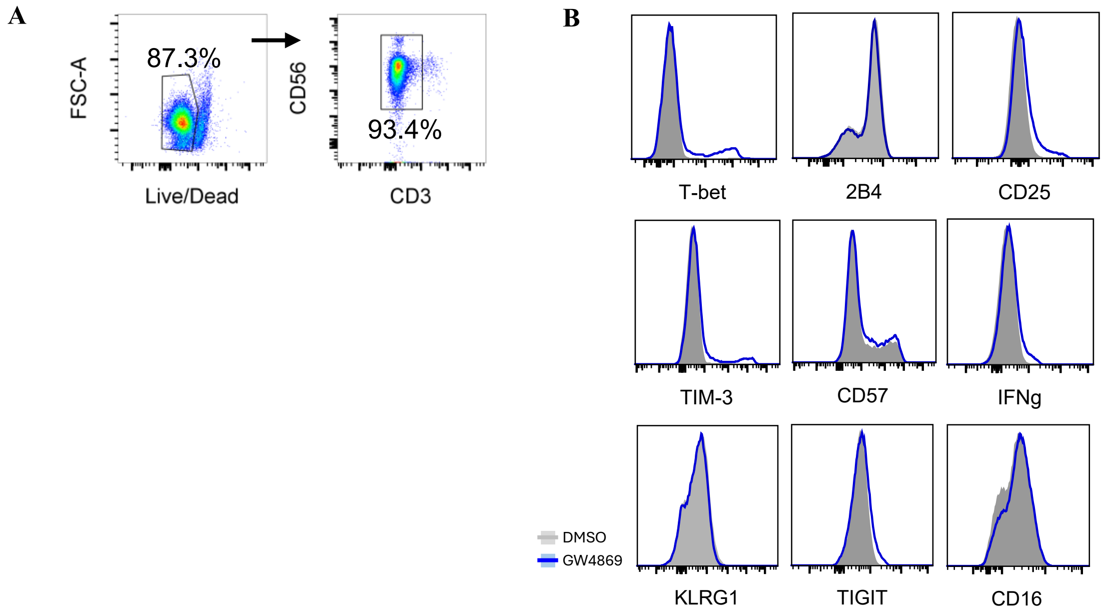

Supplementary figure 2. *(A) Representative gating strategy for NK cells. (B) Representative histograms for phenotypical changes shown in Figure 2.*

Supplementary figure 3.

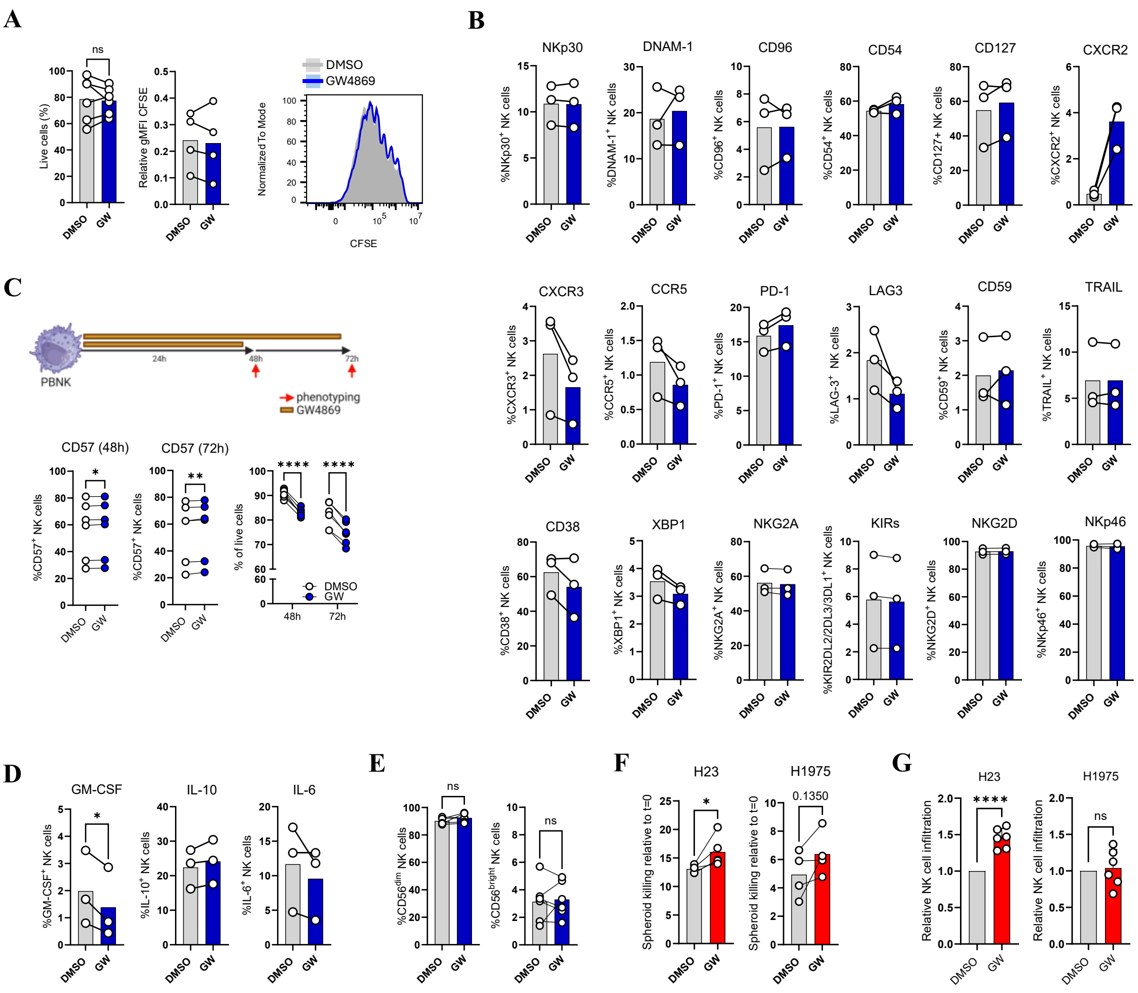

Supplementary figure 3. *(A) Viability (n=6) and proliferation (n=4) of peripheral NK cells after 24 hours or 7-day treatment with GW4869, respectively. Proliferation is relative to samples cultured in absence of IL-2. (B) Expression of surface markers in peripheral NK cells following 24h of treatment with GW4869 (n=3). (C) CD57 expression (LEFT) and viability (RIGHT) following GW4869 treatment for 48 or 72h in the absence of IL-2 (n=6). (D) Intracellular expression of GM-CSF, IL-10, and IL-6 in NK cells following 24h of treatment with GW4869 (n=3). (E) Frequency of CD56dim and CD56bright NK cells following GW4869 treatment (n=6). (F) Killing of H1975 (n=4) and H23 (n=5) spheroids measured by real-time live cell imaging at 72h. (G) Relative infiltration of GW4869-pretreated NK cells into H1975 and H23 spheroids (n=6).*

Supplementary table 1.

Supplementary table 1. *Details on antibodies and dyes used for flow cytometry*

Marker	Fluorochrome	Clone	Vendor	Cat. #	Panel
Live/Dead	NIR 775nm	/	Invitrogen	L34976	All assays
Live/Dead	Aqua	/	Invitrogen	L34957	
CFSE	FITC	/	Invitrogen	C24554	Infiltration and proliferation
GM-CSF	PE	BVD2-21C11	Biolegend	502305	Activation and maturation
CD127 (IL-7Ra)	PerCp-Cy5.5	A019D5	Biolegend	351322	
CXCR3	Alexa Fluor 700	1C6/CXCR3	BD	561320	
IL-6	PE-Cy7	MQ2/13A5	Biolegend	501119	
CCR5	FITC	HEK/1/85a	Biolegend	313705	
IL-10	PE	JES3-907	Biolegend	501403	
CD274 (PD-1)	APC	MIH4	Invitrogen	17-0969-42	
CD337 (NKp30)	PerCp-Cy5.5	P30-15	Biolegend	325215	
CD56	Alexa Fluor 700	HCD56	Biolegend	318316	
CD96 (Tactile)	PE-Cy7	NK92.39	Biolegend	338415	
CD226 (DNAM1)	PE	TX25	Biolegend	337106	
CD54	PE-Dazzle594	HA58	Biolegend	353118	
CD223 (LAG-3)	FITC	11C3C65	Biolegend	369326	
CD253 (TRAIL)	PE	RIK-2	Biolegend	308209	
T-bet	Qdot 655	O4-46	BD	564142	
XBP-1	Alexa Fluor 647	143F	Biolegend	647506	
TIGIT	Pacific Blue	MBSA43	Invitrogen	48-9500-42	
CD59	PE	P282	Biolegend	304707	
CD38	PE-Dazzle594	HB-7	Biolegend	356629	
CXCR2	APC	5E8/CXCR2	Biolegend	320710	
CD56	PE-CF594	R19-760	BD	564963	
CD3	PE-Cy5	UCHT1	Invitrogen	15-0038-42	
CD57	Pacific Blue	HCD57	Biolegend	322316	
CD16	Pacific Blue	3G8	BD	558122	
CD25 (IL-2Ra)	PE-Cy5.5	BC96	Invitrogen	35-0259-42	
KLRG1	APC/Fire 750	SA231A2	Biolegend	367717	
CD244 (2B4)	FITC	2-69	Biolegend	393510	
IFNg	Pacific Blue	B27	Biolegend	506525	
TIM-3	BV785	F3G-2E2	Biolegend	345032	
NKG2D	APC	1D11	Biolegend	345032	
NKp46	V450	9E2/Nkp46	BD	562099	
KIR2DL2/L3	FITC	DX27	Biolegend	312604	
KIR3DL1	FITC	DX9	Biolegend	312706	
NKG2A	BV650	131411	BD	747920	
MICA/B	APC	6D4	Biolegend	320908	Tumor markers
HLA-ABC	FITC	G46-2.6	BD	560965	
ULBP1	Phycoerythrin	179818	R&D systems	FAB1380P	
ULBP3	Phycoerythrin	166510	R&D systems	FAB1517P	
ICAM-1	PE	HCD54	Biolegend	322708	
